# Evaluation of *Candidatus* Liberibacter Asiaticus Efflux Pump Inhibition by Antimicrobial Peptides

**DOI:** 10.3390/molecules27248729

**Published:** 2022-12-09

**Authors:** Haoqi Wang, Nirmitee Mulgaonkar, Samavath Mallawarachchi, Manikandan Ramasamy, Carmen S. Padilla, Sonia Irigoyen, Gitta Coaker, Kranthi K. Mandadi, Sandun Fernando

**Affiliations:** 1 Biological and Agricultural Engineering Department, Texas A&M University, College Station, TX 77843, USA; 2 Texas A&M AgriLife Research & Extension Center, Texas A&M University System, 2415 E. Highway 83, Weslaco, TX 78596, USA; 3 Department of Plant Pathology, University of California, Davis, CA 95616, USA; 4 Department of Plant Pathology and Microbiology, Texas A&M University System, 2132 TAMU, College Station, TX 77843, USA; 5 Institute for Advancing Health through Agriculture, Texas A&M AgriLife, College Station, TX 77843, USA

**Keywords:** citrus greening, HLB, molecular dynamics simulation, antimicrobial peptide

## Abstract

Citrus greening, also known as Huanglongbing (HLB), is caused by the unculturable bacterium *Candidatus* Liberibacter spp. (e.g., *C*Las), and has caused a devastating decline in citrus production in many areas of the world. As of yet, there are no definitive treatments for controlling the disease. Antimicrobial peptides (AMPs) that have the potential to block secretion-dependent effector proteins at the outer-membrane domains were screened in silico. Predictions of drug-receptor interactions were built using multiple in silico techniques, including molecular docking analysis, molecular dynamics, molecular mechanics generalized Born surface area analysis, and principal component analysis. The efflux pump TolC of the Type 1 secretion system interacted with natural bacteriocin plantaricin JLA-9, blocking the β barrel. The trajectory-based principal component analysis revealed the possible binding mechanism of the peptides. Furthermore, in vitro assays using two closely related culturable surrogates of *C*Las (*Liberibacter crescens* and *Rhizobium* spp.) showed that Plantaricin JLA-9 and two other screened AMPs inhibited bacterial growth and caused mortality. The findings contribute to designing effective therapies to manage plant diseases associated with *Candidatus* Liberibacter spp.

## 1. Introduction

HLB is the deadliest disease threatening citrus production worldwide [1]. The disease is partially associated with a fastidious and unculturable bacterium *Candidatus* Liberibacter asiaticus (*C*Las), transmitted between trees by the Asian citrus psyllid *Diaphorina citri* [2,3]. There is no treatment for the disease, causing citrus trees to become unproductive and die within a few years. After its emergence in the 1920s in China, HLB widely spread to other countries in Africa and Asia. In 2005, the disease was first reported in the U.S. in South Florida. It led to an >70 percent reduction in Florida’s citrus production, resulting in economic losses of more than $8 billion [4]. HLB has now spread to many citrus-producing areas in the US, including California, Florida, Georgia, Louisiana, Puerto Rico, South Carolina, Texas, and the U.S. Virgin Islands [5].

Current management practices rely mainly on controlling the insect vector that spreads the bacteria from infected to healthy plants [6]. Since there is no single effective way to control HLB fully, many approaches have been studied, developed, and are being applied to manage the disease [6]. Some treatment strategies include a combination of broad-spectrum antibiotics, insecticides, nutritional supplements, and thermotherapy [7,8]. The foliar spray of pesticides can control the primary vector and transmitter of HLB, Asian citrus psyllids [9]. Novel RNA interference approaches targeting ACP carboxylesterase and acetylcholinesterase have very high specificity and susceptibility to the psyllids [10,11,12]. Trunk injection and foliar spraying with antibiotics such as penicillin, ampicillin, streptomycin, and oxytetracycline have been tested for their ability to suppress *C*Las populations [13,14]. Further, the U.S. Environmental Protection Agency recently approved the use of oxytetracycline [15] and the emergency use of streptomycin [16] on citrus. Furthermore, hygromycin has been deployed under a federal emergency exemption [17]. However, *C*Las strain Ishi-1 was reported to be resistant to common chemical treatments such as ampicillin and streptomycin at 1000 µg/mL [18,19]. In addition, health experts have expressed concerns about medically essential antibiotics in agriculture due to the increased risk of antibiotic resistance in humans [20,21,22,23]. Thus, there is an urgent need to find new antimicrobial agents and technologies to facilitate the rapid screening of antimicrobial agents for HLB management.

This current study screened AMPs against the TolC efflux pump protein of *C*Las to manage HLB. *C*Las is a Gram-negative bacterium with two membranes, including multiple energy-driven pumps to transport molecules from the cell interior to the extracellular space. The energy-driven pump TolC protein participates in the *C*Las secretory pathway and is embedded in the outer membrane [24]. It is proven that the *C*Las secretory pathway can be targeted to control *C*Las virulence and pathogenicity [25].

**Figure 1:**
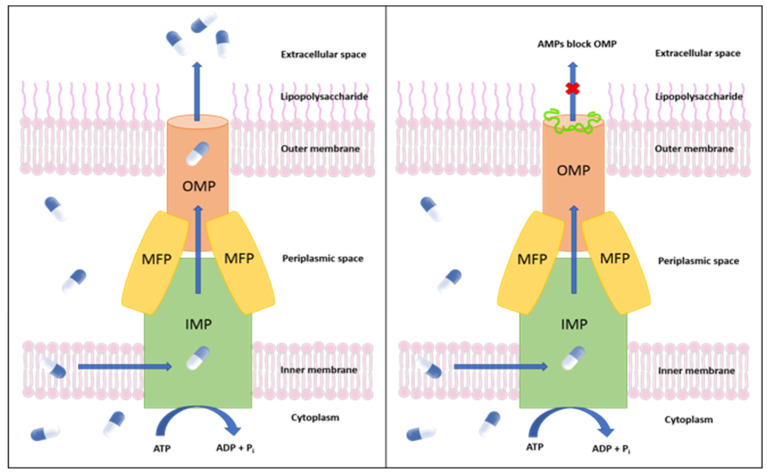
**Left:** Schematic representation of an efflux pump in *Candidatus* Liberibacter asiaticus. **Right:** Inhibition of the *C*Las TolC outer membrane protein by (AMPs). MFP: membrane fusion protein; IMP: inner membrane protein.

As with most Gram-negative bacteria, *C*Las utilizes secretion systems to transport proteins and nutrients across the membrane to manipulate host cells [26]. Some studies based on surrogate strains have shown that *C*Las-secreted proteins can regulate *C*Las multiplication and colonization of the citrus phloem cells by altering host cellular functions [27]. Blocking bacterial secretory pathways is a promising approach for novel antimicrobial discovery [28]. Whole-genome sequencing analyses reveal that *C*Las lacks the type III and type IV secretory systems and the type II plant cell wall degrading enzymes, which are known to play a critical role in the pathogenicity of Gram-negative bacteria [2,29]. *C*Las expresses all genes of type I secretory system (T1SS). The primary functions of T1SS are multidrug efflux and effector protein secretion. The T1SS is a piece of tripartite machinery composed of an inner-membrane protein (IMP) ABC transporter, a periplasmic membrane fusion protein (MFP), and TolC, an outer membrane protein (OMP) [30]. The substrates (proteins or drugs) bind to the IMP. They are transported across the inner membrane from the bacterial cytoplasm into the periplasm, using the energy produced from ATP hydrolysis. Upon substrate binding, the IMP-MFP complex associates with TolC, which induces the opening of TolC and the subsequent release of the substrate outside the bacterial cell [28]. Inhibiting the tripartite multidrug efflux pump [31] terminates the secretion of virulent effector proteins and prevents drug efflux, providing some protection against the pathogen (Figure 1).

The mechanism of how HLB *C*Las-secreted effector proteins damage the host phloem cells remains elusive [1]. A *C*Las effector SDE15 has been reported to interact with citrus papain-like cysteine proteases that break down the citrus defense system and enhance the pathogen’s virulence [25,32]. The secretion system can be targeted to control *C*Las virulence and pathogenicity.

AMPs are short sequences of amino acids. Some AMPs are known to activate the innate immune response in the host [33]. Several studies have shown that AMPs have good activity against pathogenic bacteria in plant systems [34,35]. Although studies exist on the use of AMPs and small antimicrobial molecules against *C*Las, currently, there are no studies regarding the use of AMPs for treating *C*Las through the inhibition of the bacterial secretion system [36,37,38]. This study was conducted to evaluate if select AMPs interact with the efflux pump TolC of the T1SS system to suppress the pathogenicity of *C*Las. In silico techniques were combined to virtually screen AMPs targeting the tripartite TolC efflux pump. In addition, molecular dynamics simulations and binding free energy calculations of the interactions of the complexes were performed to evaluate the screened AMPs. Using this approach, we identified multiple new AMPs that demonstrated inhibitory activity against two close culturable surrogates of *C*Las: *Liberibacter crescens* and *Rhizobium* spp. in vitro.

## 2. Results and Discussion

### 2.1. Protein Structure Modeling and Validation

Multidrug efflux proteins are resistant to many small molecules, including antibiotics. T1SS is one of the known secretion systems found in *C*Las, which can secrete offensive enzymes and effectors [39]. Finding an effective AMP to bind and inhibit the outer membrane component may be an effective way to reduce the efflux function of the entire transmembrane protein and is the strategy that was evaluated in this study.

**Figure 2:**
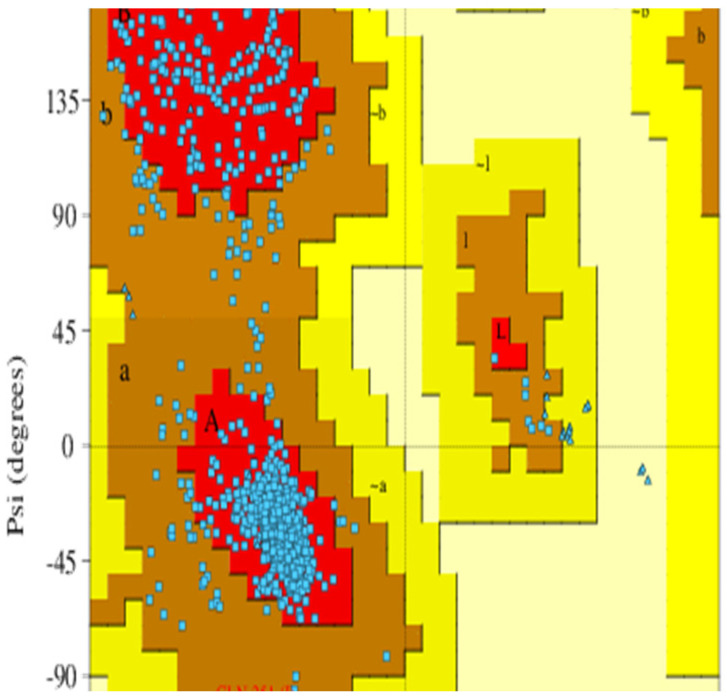
Ramachandran plot of the homology model. The red, brown, yellow, and light-yellow regions represent the most favored regions, additionally allowed regions, generously allowed regions, and disallowed regions, respectively. The residues are shown as blue dots.

Since no experimental structures were available for the *C*Las TolC protein, the TolC protein model was prepared using the homology modeling approach based on the amino acid sequence of the *C*Las TolC protein and the 3D structure of a related protein. A total of 210 efflux pump templates were found by BLAST, and the selected template had 30.16% sequence identity and 0.35 sequence similarity, as shown in Table 1 and Figure S1. The trimer homology model was built by the multidrug efflux (OMP) OprN (PDB: 5iuy) [40,41]. The ERRAT value for the homology model was 92.96, and models with an ERRAT score above 50 were considered to be of good overall quality for non-bonded atomic interactions. The passed PROVE parameter showed that no buried outlier protein atoms were found in the homology model. The VERIFY 3D parameter showed that 51.23% of the residues had an average 3D-1D score >= 0.2. This parameter was below 80%, indicating that the homology model could be optimized by further processing. The predicted homology models were first preprocessed and prepared using Schrödinger’s Protein Preparation Wizard. The trimeric TolC protein homology model consists of chains G, H, and I. Then, the Ramachandran plot model was generated by PROCHECK [42]. According to the PROCHECK report, 89.3% of the residues were in the most favored regions, 10.6% were in the additional allowed regions, 0.1% were in the generously allowed regions, and 0% were in the disallowed regions, as shown in Figure 2. The residue level in the Ramachandran plot-favored score was 99.9% (1091/1092), which met ideal conditions (>98%) [43]. This indicated that the protein backbones were favorable, and the proposed confirmation was stable. The Z score was predicted by comparison with the non-redundant PDB structure set of the proposed model (Figure S1). The Z score showed that the model was less than one standard deviation from the mean models. The predicted model was considered reliable for further in silico studies based on the overall structural assessment.

### 2.2. Virtual Screening of AMPs

Potential AMPs were screened from the APD3 database, including 2619 AMPs that were primarily natural antimicrobial peptides. The number of AMPs in the APD3 library was narrowed down by adding conditions compatible with the membrane-active peptide. Short peptide macromolecules of less than fifteen amino acids in length were selected as one of the screening criteria due to their excellent bioavailability and stability [46,47]. The AMPs were further selected based on previous evidence of inhibitory activity against bacterial efflux pumps. A preliminary screening of 15 antimicrobial peptides associated with bacterial membranes was carried out from the filter search. Eight AMPs with membrane activity against Gram-negative bacteria were further selected based on the experimental data listed in Table 2.

The AMPs were screened based on their affinities to the β-barrel structure of the TolC protein. The β-barrel structure consists of residues Ala 94 to Leu 128 and Tyr 300 to Gly 338. All the homology models of the peptide candidates had α-helical conformations and positive net charges. These screened peptides came from various sources, such as animals, insects, plants, and cultured bacteria, including temporins, urechistachykinins, colistins, plantaricin, and darobactin. Temporins are a family of antimicrobial peptides from the European red frog Rana *temporaria* [48]. These peptides were originally extracted from frog skin secretion. The tachykinin-related peptides urechistachykinin I and II are from the echiuroid worm, *Urechis unicinctus* [49]. They have known effects on neurons and G protein-coupled receptors in animals. Colistin (polymyxin E) has been used for more than 50 years to treat infections caused by Gram-positive bacteria, such as *Bacillus subtilis* [50,51]. The bacteriocin plantaricin JLA-9 is from Suan-Tsai, a traditional Chinese fermented cabbage [52]. JLA-9 has broad-spectrum antibacterial activity against Gram-positive and Gram-negative bacteria under acidic conditions. The Trp-rich peptide TetraF2W-RK is a synthetic peptide [53]. The peptide exhibits good activity targeting bacterial membranes and inhibits the antibiotic-resistant bacteria *Staphylococcus aureus* [54]. Darobactin is a newly identified antibiotic isolated from *Photorhabdus* spp. in 2019 [55]. Darobactin is effective against antibiotic-resistant Gram-negative pathogens and has low toxicity and good pharmacokinetics in vivo.

### 2.3. Molecular Docking Analysis (Glide)

The conformations with the eight AMP-protein complex docking scores were analyzed for intermolecular interactions with Standard Precision (SP) peptide docking. Table 3 shows the binding affinity of AMPs ranging from −2.787 to −9.605 kcal/mol and −7.678 kcal/mol for the efflux pump inhibitor MRL-494 as the positive control [56]. MRL-494 was found as the antibiotic targeting the outer membrane protein of bacteria. All the molecules showed multiple hydrogen bonds with the protein complex. Darobactin, with the highest binding affinity of −9.605 kcal/mol, simultaneously formed five hydrogen bonds with residues ASP314 and ASN315 on chain G, residues ASN315 and SER316 on chain H, and the ASN312 residue on chain I. In addition, eleven polar and four hydrophobic residues were proximal to darobactin at a cut-off distance of 3 Å. There were only two negatively charged residues, ASP314 on chain G and ASP314 on chain H, and no positive residues were involved in the interaction. Urechistachykinin II had a binding score of −9.332 kcal/mol and formed six hydrogen bonds. One π-π stack interaction, 13 polar residues, seven hydrophobic bonds, two negatively charged residues, and two positively charged residues were involved in the interaction of urechistachykinin II with the protein. Plantaricin JLA-9 had a binding affinity of −9.002 kcal/mol and formed six hydrogen bonds. One π-π stack interaction, one π-cation interaction, nine polar residues, four hydrophobic bonds, and two negatively charged residues were involved in the interaction of plantaricin JLA-9 with the protein. All the screened AMPs and MRL-494 interacted well with all three sub-chains of TolC simultaneously. Similar to MRL-494, darobactin, urechistachykinin, and planataricin JLA-9 formed H bonds with one or more of the residues among ASP314 on chain G and ASP314 and ASP315 on chain H, suggesting that these three peptides bind to the same domain as MRL-494.

**Figure 3:**
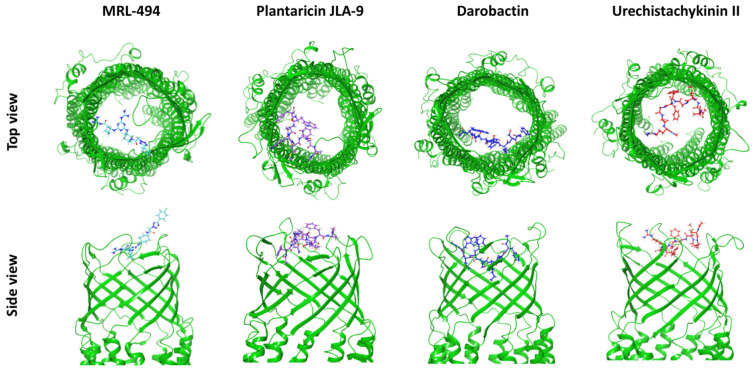
The structural snapshots of the selected AMPs interacting with TolC at the β-barrel sites. TolC efflux pump protein was shown as a green ribbon diagram. AMPs were presented via balls and stick diagrams.

Since the three AMPs, darobactin, urechistachykinin II, and plantaricin JLA-9, had a good binding affinity and close interaction with TolC, these three AMPs were selected for further kinetic simulation analyses. In Figure 3 and Figures S2–S5, the interaction diagrams of the three AMPs mentioned above showed that the docking position was at the outlet of the efflux pump. This interaction reduced the surface area at the efflux pump’s outlet to inhibit the protein’s efflux function.

### 2.4. Molecular Dynamics Simulations

**Figure 4:**
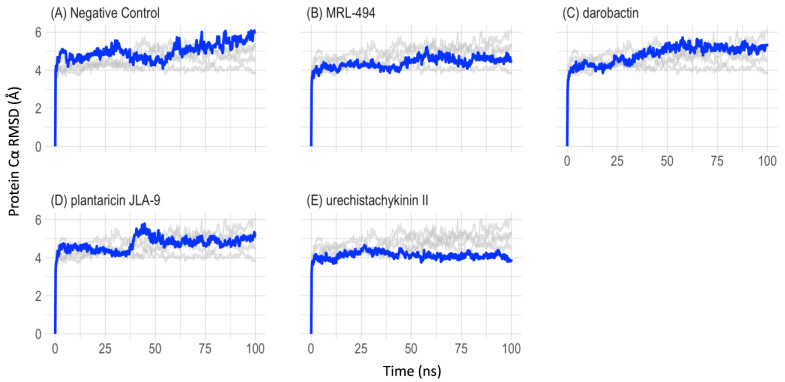
Cα RMSD for the 100 ns MD of the efflux pump complex. The main data for each graph are marked in blue, and the other four sets of data used as comparisons are marked in gray. The RMSD of all the simulated complexes of Cα was stable between 50 to 100 ns.

To understand the structural and conformational changes during peptide binding, 100 ns molecular dynamics simulations were performed for five complex systems (apo-protein, a known inhibitor, and three selected AMPs). The root-mean-square deviation of atomic positions (RMSD) and root mean square fluctuation (RMSF) were monitored to evaluate the stability of the protein and peptide complexes using the simulation trajectory. In Figure 4, the protein Cα RMSDs were compared, and the peptide-bound complexes showed a lower deviation. The average RMSD value for the overall simulation and its standard deviation of the apo-protein was 4.99 ± 0.487 Å. The average RMSD value for the overall simulation and its standard deviation of the positive control MRL-494 was 4.38 ± 0.338 Å. The average RMSD value for the overall simulations and their standard deviation of darobactin, plantaricin JLA-9, and urechistachykinin II were 4.83 ± 0.510 Å, 4.72 ± 0.407 Å, and 4.11 ± 0.258 Å, respectively. Based on the RMSD values, all these simulations were considered stable after 50 ns.

**Figure 5:**
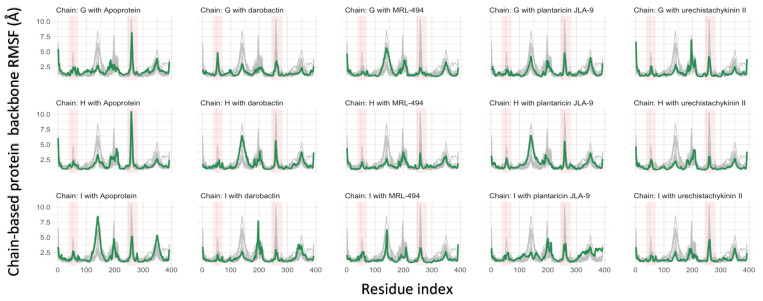
The RMSF of TolC protein. Backbone RMSFs are plotted separately (green) and compared with all the RMSFs, including apo-protein (gray) in the other plots. Active β-barrel sites of interest are highlighted in red. There are four β-sheets per chain, and each highlighted column represents two β-sheets.

In Figure 5, Cα RMSF was used to measure the flexibility and stability of the protein residues. RMSF maps were prepared for each of the three sub-chains of the trimeric efflux pump protein to view the contribution of the ligand to the residues. The residues with high RMSF values were always considered highly flexible regions. The average RMSF at the active binding region of the apo-protein was 10.4 ± 1.53 Å. The average RMSF of MRL-494 was 3.56 ± 0.513 Å. The average RMSF of darobactin, plantaricin JLA-9, and urechistachykinin II were 5.64 ± 0.810 Å, 5.51 ± 0.860 Å, and 4.62 ± 0.775 Å, respectively. All the AMP-protein complexes had lower RMSF values compared to the apo-protein, suggesting that AMP binding can form stable protein-AMP complexes.

### 2.5. Prime MM-GBSA Analysis

The molecular mechanics-generalized Born surface area (MM-GBSA) binding energy method was performed to calculate the binding energies between the AMPs and the TolC protein quantitatively. Table 4 presents the overall average binding free energy (ΔG_bind_) and the specific binding free energy components. All the listed energies were obtained and calculated from the last 50 ns of the coordinate sampling of the simulated trajectory. The ΔG_bind_ of MRL-494 was −63.55 ± 8.51 kcal/mol. The ΔG_bind_ of darobactin, plantaricin JLA-9, and urechistachykinin II were −42.09 ± 11.20 kcal/mol, −51.84 ± 10.90 kcal/mol, and −46.15 ± 12.09 kcal/mol, respectively. The ligand strain energy represents the energy cost of getting the compound into the active pocket. This is the energy difference between the optimized drug–protein complex and the drug outside the binding pocket. The ligand strain energies for the screened peptides, in increasing order, were plantaricin JLA-9, darobactin, and urechistachykinin II. Lower ligand strain energies are associated with a ligand more easily accommodating and fitting to its associated active site.

### 2.6. Principal Component Analysis (PCA)

**Figure 6:**
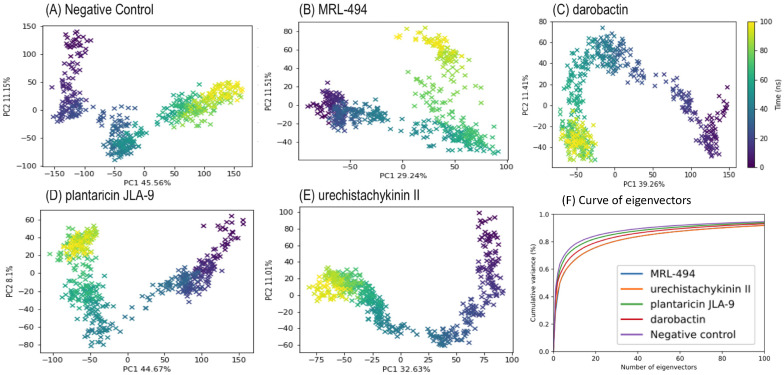
PCA on Cα atoms from the MD simulation constructed by the first two eigenvectors, PC1, and PC2. (**A**) Apo-protein (Negative control). (**B**) MRL-494. (**C**) Darobactin. (**D**) Plantaricin JLA-9 (**E**) Urechistachykinin II. (**F**) explained variance curves of the first 100 principal components for these five simulations.

To understand the motion of the simulation, a PCA was performed using the pairwise distance method. Five hundred frames were sampled to represent the changes across the simulation (Figure 6A–E). The first two eigenvectors, PC1 and PC2, in all five PCA tests contributed to approximately 50% of the variance to the motion. During the MD simulation, the conformational changes of the docked peptide ligands and protein receptors were represented by a color gradient from purple to yellow. Figure 6F shows that the first few eigenvectors explained most of the variance, and when the number of eigenvectors reached 100, it explained practically 90% of the total variance. The PC1 vs. PC2 scatter plots explained the structural changes by the AMP interactions. The graphs showed consecutive point changes, and no outliers were found, indicating a smooth simulation. The PC1 of apo-protein exhibited a higher range (~300) than the interacting complexes (~200). This phenomenon also occurred on the apo-protein’s PC2. The PCA’s clustering of the data points suggested structural stabilization, whereas scattering suggested structural changes. According to the first and second principal components, the interacting complexes had low structural variation. According to the last 50 ns of the simulation, all the AMP groups underwent a point of cluster distribution, which was thought to reduce protein flexibility due to binding, thus limiting the variability of protein conformation. These AMP-protein structures also hindered the cylindrical outlet, reducing or even blocking the enforced excretion of intracellular drugs.

### 2.7. Biological Efficacy Assays

Because *C*Las is unculturable, the biological efficacy of the three in silico predicted peptides was assessed using two closely related surrogates, a culturable *Rhizobium galegae* as well as a moderately culturable *Liberibacter crescens*. Such surrogate models are related to *C*Las and have been used for the potential drug screening and testing of uncultured Liberibacter [9,13]. For example, a surrogate *Sinorhizobium meliloti* was used to study the effects of *C*Las transcription regulator proteins LdtR, RpoH/RpoH1, and VisNR [57,58,59]. *C*Las SecA protein was studied in silico and in vitro for potential small molecule inhibitors using *L. crescens*. *L. crescens* was used to study the mode of action of the transcriptional accessory protein PrbP in interactions with tolfenamic acid [60,61]. In this study, the surrogate bacteria *R. galegae* and *L. crescens* were closely related to *C*Las, and in the same order as *Rhizobiales.*

According to the Tblastn sequencing analysis, the *C*Las efflux pump protein aligned well with corresponding homologs in the culturable surrogates [62]. In *R. galegae*, the aligned segments were in nucleotides 4,025,395 to 4,026,762 and 2,135,053 to 2,135,241. The first segment covered 98% of the *C*Las TolC protein, and the second covered 16% of the *C*Las TolC protein. The first segment aligned with *C*Las. The identity rate was 42%. The max score was 382, with an E value of 9 × 10^−120^ In *L. crescens*, the aligned segment was in nucleotides 1,077,242 to 1,078,594. The aligned sequences showed 98% coverage of *C*Las proteins and 48% identity. The max score was 416, with an E value of 9 × 10^−132^. The results indicated that the surrogates had similar functional proteins associated with the *C*Las TolC efflux pump protein.

**Figure 7:**
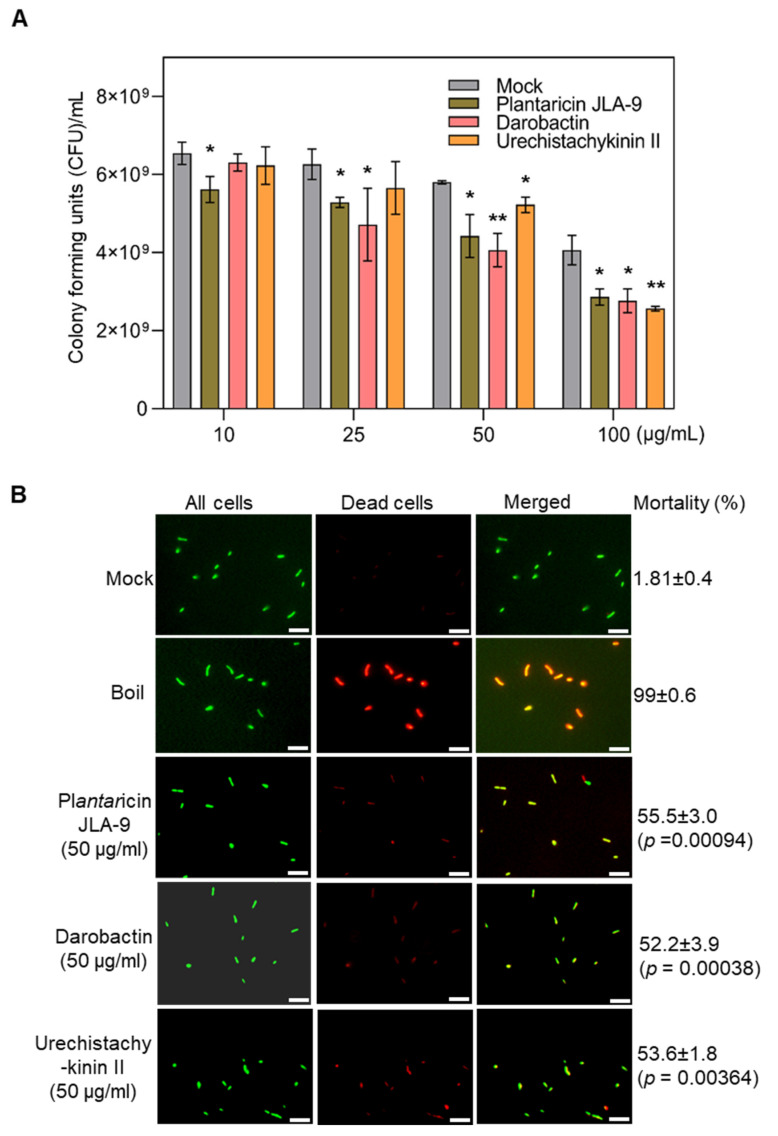
Efficacy of Plantaricin JLA-9, Darobactin, and Urechistachykinin II against *R. galegae*. (**A**) *R. galegae* colony-forming units (CFU)/mL were calculated after treatment with Plantaricin JLA-9, Darobactin, and Urechistachykinin II at multiple dosages (10, 25, 50, and 100 µg/mL) along with an equal concentration of solvent DMSO (0.6, 1.2, 2.4% *v*/*v*) used for dissolving the peptides as a mock control. Asterisks indicate statistically significant differences between control and treatment by Student’s *t*-test (*, 0.01 and **, 0.001). (**B**) *R. galegae* cell viability/mortality was estimated after treatment with AMPs (50 µg/mL) and along with a mock (DMSO, 2.4% *v*/*v*). Boiled cells were used as a positive control. Percent (%) mortality was estimated by counting the number of dead cells (red stained) among all cells (green stained). Error bars represent ± standard error of the mean (*n* = 5). *p*-values were calculated by a two-sample *t*-test (one-tailed) relative to mock control. Scale bar = 10 µm.

The efficacy of the peptides on *R. galegae* was examined by quantifying the growth of bacteria, i.e., colony forming units (CFU) upon treatment with the AMPs. In these assays, Plantaricin JLA-9 significantly inhibited *R. galegae* (*p* ≤ 0.05) in all the tested dosages (10, 25, 50, and 100 μg/mL) when compared to the mock control. Darobactin significantly (*p* ≤ 0.05) inhibited *R*. *galegae* at 25, 50, and 100 μg/mL, whereas Urechistachykinin II showed efficacy only at 100 μg/mL (Figure 7A). In addition, a viability/mortality assay was performed using the viability/cytotoxicity assay kit (Biotium) that uses two fluorescent dyes to detect dead bacteria as well as all bacteria. *R. galegae* were treated with the three peptides at a dosage of 50 μg/mL and cell mortality rates were estimated by counting the number of dead cells (red stained) among all cells (green stained) (Figure 7B). All three peptides, Plantaricin JLA-9, Darobactin, and Urechistachykinin II caused significant mortality of 55.50, 52.20, and 53.60%, respectively, thus confirming the antimicrobial activity against *R. galegae* (Figure 7B). Similarly, efficacy of the three peptides was assayed on *L. crescens* using the viability/cytotoxicity assay. Plantaricin JLA-9 and Urechistachykinin II showed significant cell mortality (*p* ≤ 0.01); however, Darobactin did not impact *L. crescens* viability at the concentrations tested, suggesting bacteria-specific differences (Supplemental Figure S6).

In summary, the in vitro bioassays indicated that Plantaricin JLA-9, Darobactin, and Urechistachykinin II showed inhibitory activities in one or both surrogate bacteria. Plantaricin JLA-9 showed significant inhibition of *R. galegae* as low as 10, 25, 50, and 100 µg/mL and *L. crescens* at 50 and 100 µg/mL. Plantaricin JLA-9, initially isolated from Suan-Tsai, showed broad-spectrum antibacterial activity against Gram-positive and Gram-negative bacteria [52]. Darobactin showed significant inhibition of *R. galegae* at 25, 50, and 100 µg/mL. Darobactin is a bicyclic peptide discovered in *Photorhabdus* bacteria in 2019 [55]. In this study, darobactin was synthesized as a linear peptide; thus, the bioactivities could be different. Urechistachykinin II showed inhibition of *R. galegae* at 50 and 100 µg/mL, whereas *L. crescens* showed mortality at 100 µg/mL. Urechistachykinin II is a tachykinin-related neuropeptide from the echiuroid worm with bioactivity on neurons and G protein-coupled receptors in animals [49]. In other studies, Blacutt identified three active natural compounds, cladosporols A, C, and D, using the *L. crescens* diffusion assay [63]. These three compounds were found to have dose-dependent antagonisms to *L. crescens.* Zhang screened two small bioactive molecules (P684-2850, P684-3808) with strong antimicrobial activities to *L. crescens* in vitro [60]. The IC50 values for P684-2850 and P684-3808 were 11 µg/mL and 15 µg/mL, respectively. Although our surrogate bioassays verified the bioactivities of the in silico screened molecules, further long-term structure–function relationships and *in planta* efficacy trials are necessary to understand the inhibitory mechanism on *C*Las.

## 3. Materials and Methods

### 3.1. Sequence Analysis

Computational predictions of the three-dimensional structures of the proteins were constructed using comparative homology modeling techniques. The *C*Las TolC efflux pump protein was identified by the NCBI unique identifier CLIBASIA_04145 in *C*Las [2]. Based on the SWISS-MODEL server [64], the crystal structure of the efflux pump component OprN from *Pseudomonas aeruginosa* (PDB: 5iuy) [40] was used as a template to construct a structural model of the *C*Las TolC efflux protein. The Ramachandran plots were visualized by PROCHECK [42] to evaluate the effectiveness of the homology model(s). The overall quality of the homology models was evaluated by ERRAT [65], VERIFY 3D [66,67], and PROVE [68] on the SAVES v6.0 server (https://saves.mbi.ucla.edu accessed on 1 October 2022).

### 3.2. Virtual Screening of AMPs

The initial set of AMPs was screened from the Antimicrobial Peptide Database (APD3) [69]. Several criteria were considered for screening AMPs targeting the membrane protein tripartite efflux pump in *C*Las. First, the AMPs should be active against Gram-negative bacteria. Second, the sequence length of the AMPs was around 15 amino acids to allow stability and bioavailability. Additionally, AMPs with known membrane protein activities were also included. All these parameters were applied in the search engine in APD3.

### 3.3. Molecular Docking

The binding mode prediction of AMPs with the *C*Las efflux pump receptor complex was performed on the Schrödinger Glide platform [70]. Both the protein and AMPs were prepared by Schrödinger’s Protein Preparation Wizard [71]. The AMPs were docked on the rigid protein receptor to find the potential binding pockets with the highest docking scores. The ligand-receptor interactions were visualized using Schrödinger Maestro [72], and the AMPs with multiple and stable non-covalent interactions were selected for molecular dynamics (MD) simulations. MRL-494, a small-molecule inhibitor of β-barrel assembly machine A, was used as the positive control [56].

### 3.4. Molecular Dynamics Simulations

Promising AMPs from the initial docking studies were selected to elucidate the possible mode of action (MOA) in terms of protein–peptide binding interactions [73]. The MD simulation was performed on the Schrödinger Desmond platform [73]. The system charge was neutralized with Na or Cl ions and a 0.1 M concentration of NaCl. The system was built with the Simple Point-Charge (SPC) solvent model. The boundary conditions were set as an orthorhombic box with a buffer distance of 10 Å. The model membrane of the system was the bilayer 1-palmitoyl-2-oleoyl-sn-glycerol-3-phosphocholine (POPC) membrane. The protein–ligand complex was prepared by Schrödinger’s Protein Preparation Wizard [71]. The docked AMPs were exported from Glide. The C and N termini of the protein were capped to stabilize the protein structure. All the missing hydrogens were added, and the hydrogen bonds were optimized. The strained minimization was performed with the OPLS3e force field [74].

Each simulation was performed with Desmond’s default relax protocol. The force field for the simulation was also OPLS3e. The system-heavy atoms were first minimized with restraints under 10 K, then increased in temperature to 300 K with restraints, and the final relaxation step under 300 K Normal Pressure and Temperature (NPT) ensemble to obtain the equilibrium status for the system. After relaxation, the simulations were performed under a 300 K NPT ensemble at 1.01325 bar pressure for 100 ns. The recording interval was 200 ps, and 500 frames were saved. Post-simulation trajectory analyses, including complex (RMSD) and ligand/protein (RMSF) complex interactions were performed by Schrödinger Simulation Interactions Diagram.

### 3.5. Calculation of Binding Free Energy

The binding interactions of peptide–protein complexes can be calculated using (MM-GBSA) binding energy method [75]. The primary MM-GBSA uses the VSGB 2.0 dissolution model with the OPLS3e force field.

The docking poses of the AMPs on the receptor were evaluated by calculating the total binding free energy using Schrödinger Prime. Prime MM-GBSA calculations gave the complex binding energies, which validated the performance of the current binding conformation. The Schrödinger Prime calculates the energy of the AMP-protein system via MM-GBSA using the Desmond simulation trajectory. From the entire 100 ns simulation, the last 50 ns trajectory was chosen for the energy calculation. The free energy of binding ΔG_bind_ was calculated as the energy of the receptor-ligand complex minus the energy of the receptor alone and the ligand alone, as follows:

(1) $${\Delta G}_{\mathrm{bind}}=E_{\mathrm{complex}\left(\mathrm{minimized}\right)}-\left(E_{\mathrm{ligand}\left(\mathrm{minimized}\right)}+E_{\mathrm{receptor}\left(\mathrm{minimized}\right)}\right)$$

### 3.6. Principal Component Analysis

To study the overall motion of the protein system throughout the entire simulation trajectory, a principal component analysis (PCA) for a pairwise distance of alpha carbon atoms (Cα) was performed. Visual Molecular Dynamics (VMD) [76] software was used to prepare the topology and trajectory files. The python package MDTraj [77] was used for analysis and visualization. Cα was extracted from 500 snapshots of the 50 ns MD trajectories to construct the structure matrix. Every snapshot was aligned to the starting frame before constructing the eigenvectors. The first two principal components (PC1 and PC2) were projected to represent the protein dynamics in the MD trajectories of the AMP-protein and the apo-protein systems.

### 3.7. Peptide Synthesis

The three in silico predicted peptides (Plantaricin JLA-9, Darobactin, and Urechistachykinin II) were synthesized using PeptideSyn^TM^ Peptide Synthesis Technology (LifeTein, LLC, Somerset, NJ, USA). The purity of the peptides was >95%, as determined by High-Performance Liquid Chromatography (HPLC) and Mass Spectrometry (MS) (Supplementary Dataset). All the peptides were dissolved in 100% DMSO and diluted further for in vitro efficacy testing.

### 3.8. Biological Efficacy Assays

Because *C*Las is unculturable, the biological efficacy of the three peptides was determined in vitro using closely related surrogates, *Rhizobium galegae* and *Liberibacter crescens*. The sequence analysis and comparison of the *C*Las TolC efflux protein CLIBASIA_04145 and surrogate hosts *R*. *galegae* and *L*. *crescens* were performed using Tblastn [78]. For *R*. *galegae* assays, cells were grown at 28 °C in YM broth (Yeast extract 3 g/L, Malt extract 3 g/L, Dextrose 10 g/L, Peptone 5 g/L, pH 6.2) to a cell density (OD_600_) of 1.0. Next, the *R. galegae* cells were diluted into a fresh YM media to a cell density of 1 × 10^6^. Multiple concentrations of the three peptides (10, 25, 50, and 100 μg/mL) were mixed in a 96-well plate (Nunc F96 microtiter plate) with a bacterial suspension (at a final concentration of 1 × 10^6^ colony forming units [CFU] per ml for bacteria) to a total volume of 100 µL. Three biological replicates for each concentration and peptide were used. Because pure DMSO was used to dissolve the peptides, a mock control containing equivalent concentrations of final DMSO concentration (0.2, 0.6, 1.2, and 2.4% *v*/*v*), corresponding to the different peptide dosage (10, 25, 50, and 100 μg/mL), was also included. The cells were treated by incubation at 28 °C for 2 h, and then 50 µL was plated on YM agar plates. Subsequently, the plates were incubated at 28 °C for 48 h and CFUs were estimated.

*L. crescens* is still a moderately fastidious bacterium and generally forms a lawn instead of colonies. Hence, we conducted a cell viability/mortality assay using dyes that distinguishes between live and dead cells (Biotium, Fremont, CA, USA). Selected dosages of AMPs (50 and 100 μg/mL) were tested on *L. crescens* as well as *R. galegae*. Briefly, ~3 mL of *L. crescens* culture was grown in a BM7 (α-Ketoglutarate 2 g/L, ACES buffer 10 g/L, KOH 3.75 g/L, Fetal Bovine Serum 15%, TMN-FH Medium 30%, pH 6.8) medium for 4 days. The cells were harvested by centrifuging 1 mL of cultures at 7000× *g*. The cells were washed three times with 0.85% NaCl and diluted to OD600 of 0.1 with 0.85% NaCl. The *R. galegae* cells were grown overnight at 28 °C, 200 rpm in a 3 mL TY medium. All cells were treated further with antimicrobial peptides at 50 or 100 µg/mL for 3 h at 28 °C in a plate shaker at 280 rpm. Cells were collected from the microplate into a 1.5 mL tube and centrifuged at 10,000× *g* for 10 min, the supernatant was discarded, and cells were resuspended with 100 µL of 0.85% NaCl. As a control treatment, cells were collected and boiled for 5 and 10 min for *R. galegae* and *L.crescens*, respectively. After treatment, 1 µL of dye mix was added to each treatment and incubated at room temperature for 15 min in the dark. Cells were imaged using an Olympus BX51 (Olympus LifeScience, Waltham, MA, USA) fluorescence microscope under a 60× (Immersion oil) objective using FITC and TRITC filter sets. Representative images were taken, and the percent mortality was calculated from an average of 6 field of views from each treatment.

## 4. Conclusions

In this study, we screened AMPs as potential inhibitors for *C*Las by blocking TolC, a key protein in the efflux pump of the T1SS system. Multiple in silico approaches, such as homology modeling, molecular docking, MD simulations, MM-GBSA calculations, and PCA were used to uncover potential AMPs that target the outer membrane protein TolC. Initial docking studies revealed three AMPs with good binding affinities to TolC. MD simulations for 100 ns confirmed the ability of the AMPs to bind tightly and interact with TolC receptors. Based on further PCA and Gibbs free energy calculations, plantaricin JLA-9 showed the ability to block the β barrel entrance of the TolC protein. The in vitro bioassay indicated that Plantaricin JLA-9, Darobactin, and Urechistachykinin II showed inhibitory activities in one or both surrogate bacteria. Plantaricin JLA-9 showed significant inhibition even at a lower concentration to *R. galegae* at 10 µg/mL; darobactin showed significant inhibition of *R. galegae* at 25, 50, and 100 µg/mL, and urechistachykinin II showed inhibition at 50 and 100 µg/mL. In the case of *L. crescens*, the Plantaricin JLA-9 and Urechistachykinin II showed significant mortality at 50 and 100 µg/mL. In conclusion, the strategy and results from this study could help design effective therapies to manage plant diseases caused by *Candidatus* Liberibacter spp.

## Figures and Tables

**Table 1:** Homology models generated using SWISS-MODEL.

*Template*	*Chain*	*GMQE*	*QSQE*	*Seq Identity*	*Seq Similarity*	*Membrane Protein*	*Refs.*
5iuy	A	0.71	0.51	0.30	0.35	OprN	[40]
4mt0	A	0.71	0.47	0.26	0.33	MtrE	[44]
3pik	A	0.71	0.43	0.24	0.32	CusC	[45]

**Table 2:** Information on the screened AMPs. All peptides are effective against Gram-negative bacteria.

No.	Peptide	Anti-Gram-Negative	Definition ^a^	APD3 ID	Length	Hydrophobic Residue%	Net Charge
**1**	LSPNLLKSLL	No	Temporin H (linear)	AP00859	10	50	2
**2**	LRQSQFVGSR	Yes	Urechistachykinin I (linear)	AP01480	10	30	3
**3**	AAGMGFFGAR	Yes	Urechistachykinin II (linear)	AP01481	10	60	2
**4**	KTKKKLLKKT	Yes	Colistin A (cyclic)	AP02204	10	20	6
**5**	FLPLIGRVLSGIL	Yes	Temporin A (linear)	AP00094	13	61	2
**6**	FWQKMSFA	Yes	Plantaricin JLA-9 (linear)	AP02677	8	62	1
**7**	WWWLRKIW	Yes	TetraF2W-RK (linear)	AP02856	8	75	3
**8**	WNWSKSF	Yes	Darobactin (bicyclic)	AP03168	7	42	1

^a^ The peptides modeled in this study were generated using linear amino acid sequences. The structures of the peptides tested may not be identical to the known AMPs.

**Table 3:** AMPs screened by APD3 and the Standard Precision (SP)-peptide mode docking score and interactions with the receptor. The selection of residue groups located within 3 Å of the AMP ligand.

Definition	Docking Score	Glide Model	H-Bond	π-π Stack	π-Cation
**Temporin H**	−8.971	−120.362	G: ASP314; H: ASN315, SER316; I: ASN315, SER316		
**Urechistachykinin I**	−8.610	−88.383	G: ASP314, ASN315; H: ASP314, ASN315, SER316; I: SER316		
**Urechistachykinin II**	−9.332	−146.077	G: LYS113, HIE313; H: ASN315, SER316, PHE317; I: SER316;	I: PHE317	
**Colistin A**	−7.325	−45.045	G: ASP314, SER316, PHE317, TYR320; H: ARG107, ASP314; I: SER316		G: TYR320
**Temporin A**	−2.787	2.406	G: ASP314, ASN315		
**Plantaricin JLA-9**	−9.002	−109.183	G: HIE313, ASP314; H: ASP314, ASN315, SER316; I: SER316	I: TYR320	H: ASP314
**TetraF2W-RK**	−8.700	−122.832	G: ASP314, ASN315, ASN321; I: SER316	G: PHE317	
**Darobactin**	−9.605	−144.059	G: ASP314, ASN315; H: ASN315, SER316; I: ASN312		H: ASP314, PHE317
**MRL-494**	−7.678	−66.449	G: ASP314; H: ASP314, ASN315;		

**Table 4:** AMPs screened by APD3 and the SP-peptide mode docking score and interactions with the receptor.

Compound Name	ΔG_bind_ Overall (kcal/mol)	ΔG Coulomb Energy (kcal/mol)	ΔG Covalent Binding Energy (kcal/mol)	ΔG Lipophilic. Energy (kcal/mol)	ΔG Van der Waals Energy (kcal/mol)	ΔG Generalized Born Electrostatic Solvation Energy (kcal/mol)	ΔG Ligand Strain (kcal/mol)
Darobactin	−42.09 ± 11.20	−7.78 ± 40.00	1.49 ± 4.29	−8.10 ± 4.60	−48.49 ± 7.58	23.70 ± 35.44	17.69 ± 9.98
Plantaricin JLA-9	−51.84 ± 10.90	−8.24 ± 14.95	1.34 ± 3.48	−13.09 ± 3.39	−46.83 ± 10.72	18.40 ± 12.91	10.30 ± 6.76
Urechistachykinin II	−46.15 ± 12.09	−19.16 ± 24.41	−1.27 ± 4.50	−13.88 ± 2.79	−48.25 ± 8.78	39.78 ± 20.54	29.48 ± 5.98
MRL-494	−63.55 ± 8.51	−39.08 ± 26.15	5.64 ± 2.20	−12.55 ± 1.97	−43.96 ± 4.64	34.24 ± 25.02	7.35 ± 2.88

## Data Availability

The protein–ligand interaction data and whole Desmond simulation reports are available in the GitHub repository. (https://github.com/sfernando-BAEN/AMP_project accessed on 3 March 2021). The Schrödinger drug discovery platform version 2018-4 was used in this study. (https://www.schrodinger.com/drug-discovery accessed on accessed on 3 March 2021). On this platform, Desmond used an academic license. The Protein Preparation Kit, Glide, and Prime used a commercial license. MDTraj package version 1.9.4 was performed in the python 3 environment (https://mdtraj.org/1.9.4/index.html accessed on 3 March 2021). The RMSD and RMSF plots were prepared by ploty package version 4.9.2.1 with R version 4.0.2 (Montreal, QC, Canada).

## Supplementary Materials

The following supporting information can be downloaded at: https://www.mdpi.com/article/10.3390/molecules27248729/s1, Figure S1. Homology modeling results. Figure S2. Interactions between MRL-494 (positive control) and receptors under SP-peptide docking mode. Figure S3. Interactions between darobactin and receptors under SP-peptide docking mode. Figure S4. Interactions between plantaricin JLA-9 and receptors under SP-peptide docking mode. Figure S5. Interactions between urechistachykinin II and receptors under SP-peptide docking mode. Figure S6. Efficacy of antimicrobial peptides against *L. crescens* viability and its mortality. Table S1. The detailed information of the screened AMPs. And AMP purity by HPLC in supplementary.pdf.
