# Supplementary material for: Evaluation of Candidatus Liberibacter Asiaticus Efflux Pump Inhibition by Antimicrobial Peptides
Source: Molecules. 2022 Dec 9;27(24):8729. doi: 10.3390/molecules27248729 (PMC9782701; doi:10.3390/molecules27248729)
Supplement: Supplementary file 1 [file molecules-27-08729-s001.zip › molecules-1984843-supplementary.pdf]

Supplementary data

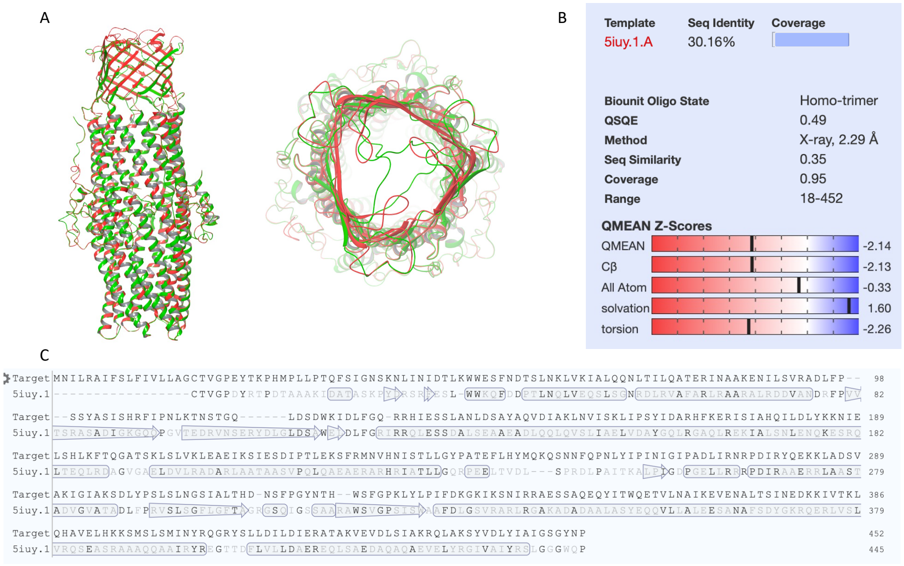

Figure S1. Homology modeling results. A) The side and top view of the homology model (green) overlaid on the template (red). B) The sequence similarity, identity information and the QMEAN Z-Scores for the model. The QMEAN score for this model is -2.14. Models of low quality typically have scores of -4.0 or lower. C) The sequence alignment of the homology model and the template.

MRL-494

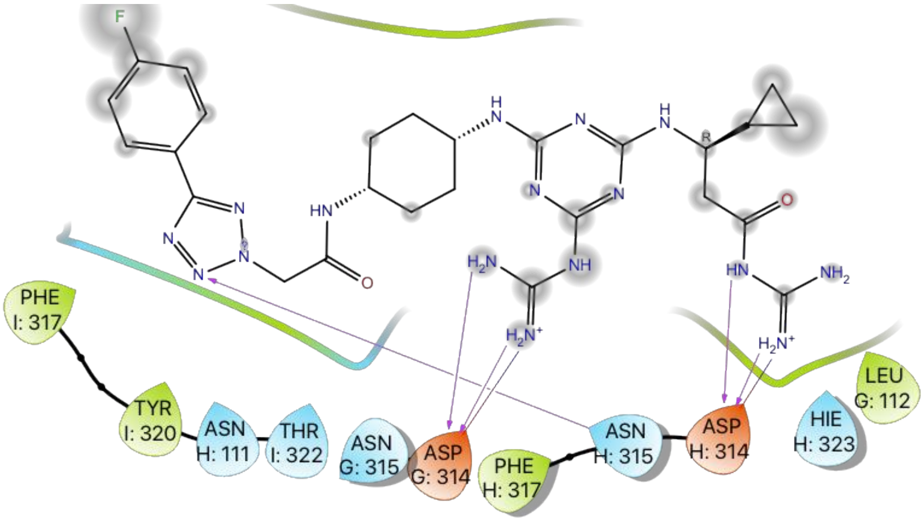

Figure S2. Interactions between MRL-494 (positive control) and receptors under SP-peptide docking mode.

Darobactin

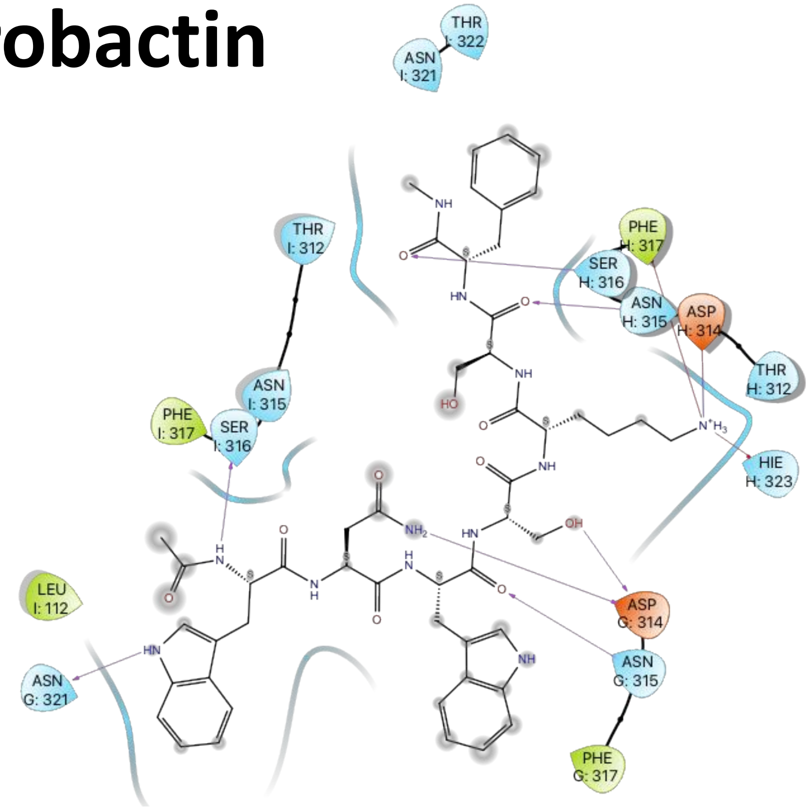

Figure S3. Interactions between darobactin and receptors under SP-peptide docking mode.

Plantaricin JLA-9

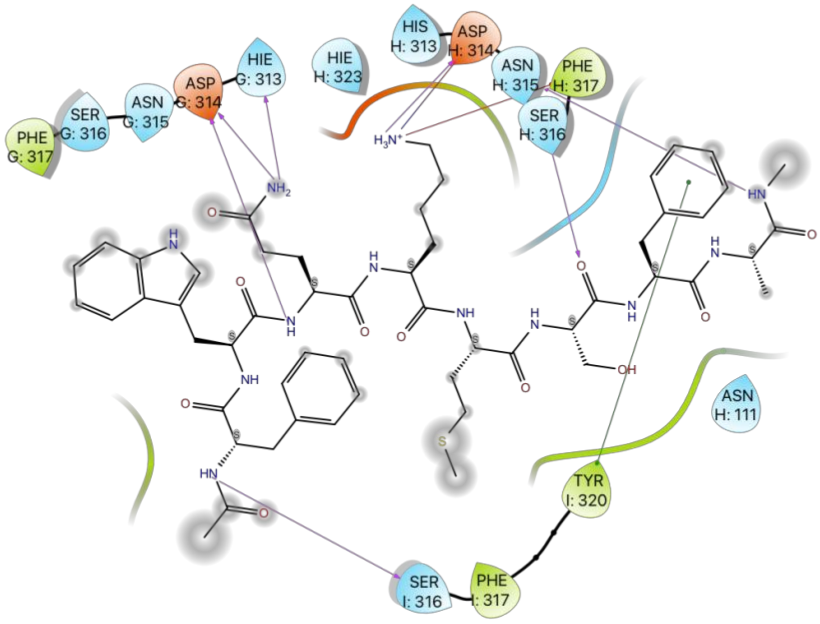

Figure S4. Interactions between plantaricin JLA-9 and receptors under SP-peptide docking mode.

Urechistachykinin II

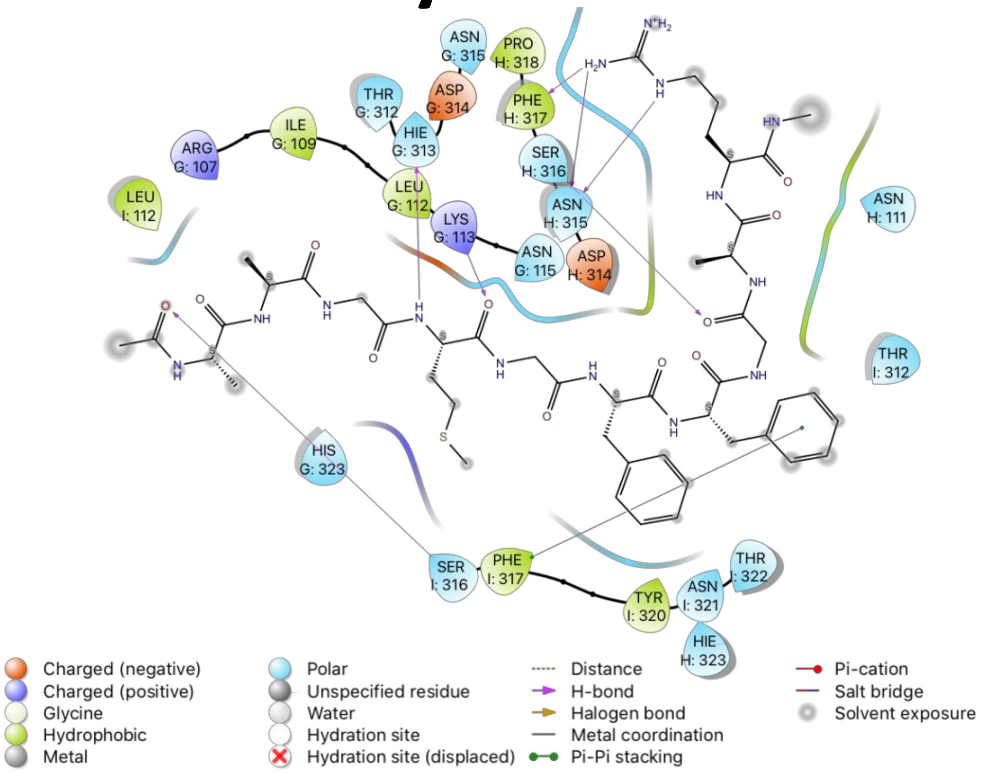

Figure S5. Interactions between urechistachykinin II and receptors under SP-peptide docking mode.

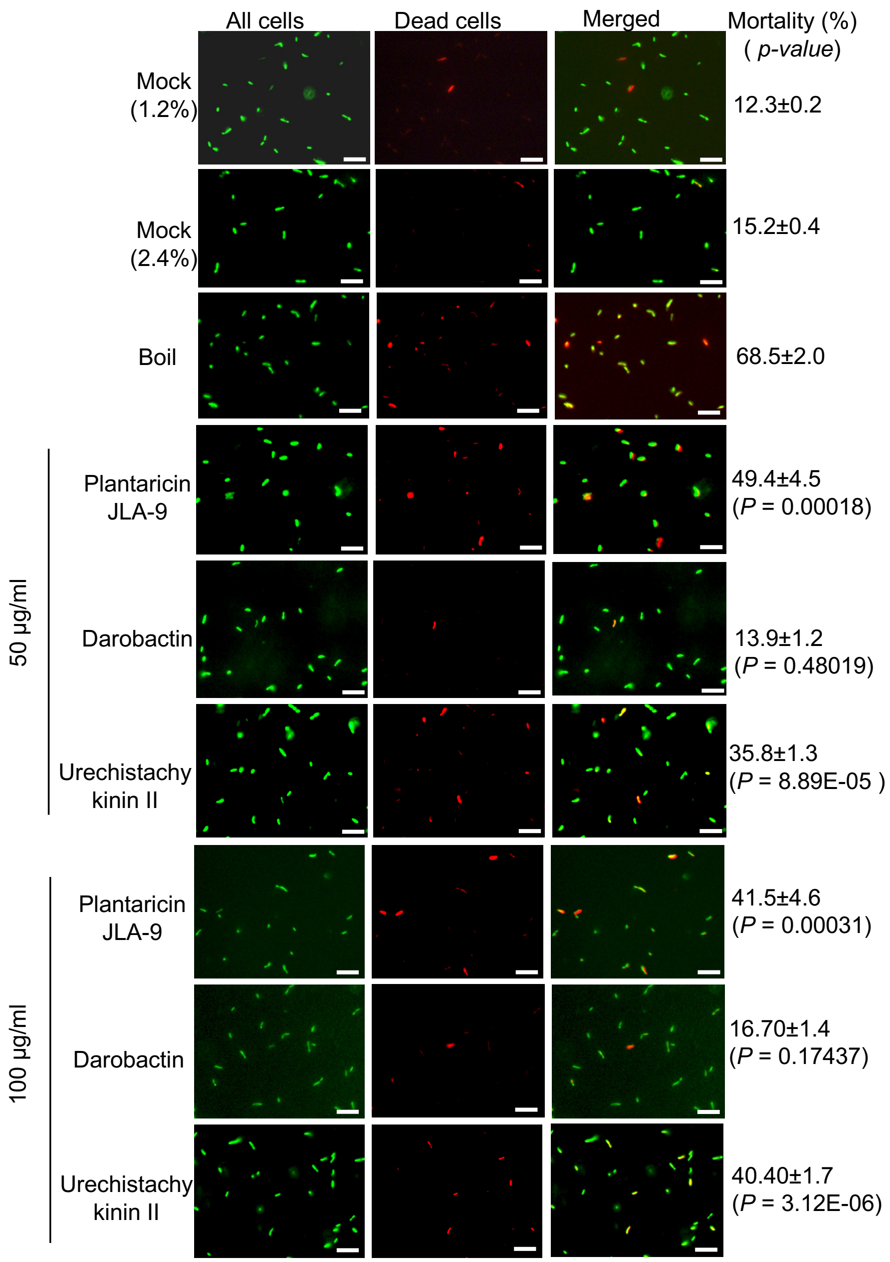

Figure S6. Efficacy of antimicrobial peptides against *L. crescens* viability and its mortality. Mortality of *L. crescens* treated with Plantaricin JLA-9, Darobactin, and Urechistachykinin II. Two dosages of the three peptides (50 and 100 µg/mL) were tested alongside mock control with an equal concentration of DMSO (1.2 and 2.4% v/v) used to dissolve the peptides. Boil treatment was used as a positive control. Percent (%) mortality was estimated by counting the number of dead cells (red stained) among all cells (green stained). Error bars represent ± standard error of the mean (n=5). p-values were calculated by a two-sample t-test (one-tailed) relative to mock control. Scale bar = 10 µm.

Table S1. The detailed information of the screened AMPs. All peptides have been shown to be effective against Gram-negative bacteria.

No	Peptide	Definition	Docking Score	glide emodel	H-bond	π-π stack	π-cation	Polar	Hydrophobic	Negative charged	Positive charged	Glycine
1	LSPNLLKSLL	Temporin H	-8.971	-120.362	G: ASP314; H: ASN315, SER316; I: ASN315, SER316			G: ASN315, ASN321, HIS323; H: HIS313, ASN315, SER316, HIE323; I: ASN315, SER316	G: LEU112; H: PHE317; I: ILE109, LEU112, PHE317, PRO318, TYR320	G: ASP314; H: ASP314	I: LYS113	
2	LRQSQFVGSR	Urechistachykinin I	-8.610	-88.383	G: ASP314, ASN315; H: ASP314, ASN315, SER316; I: SER316			G: ASN115, HIE313, ASN315, ASN321, HIS323; H: ASN111, ASN315, SER316, HIE323; I: ASN316, ASN321, THR322	H: PHE317; I: LEU112, PHE317, PRO318, TYR320	G: ASP314; H: ASP314	G: LYS113; H: LYS113	
3	AAGMGFFGAR	Urechistachykinin II	-9.332	-146.077	G: LYS113, HIE313; H: ASN315, SER316, PHE317; I: SER316;	I: PHE317		G: ASN115, THR312, HIE313, ASN315, HIS323; H: ASN111, ASN315, SER316; I: THR312, ASN312, SER316, THR322, HIE323	G: LIE109, LEU112; H: PHE317, PRO318; I: LEU112, PHE317, TYR320	G: ASP314; H: ASP314	G: ARG107, LYS113	
4	KTKKKLLKKT	Colistin A	-7.325	-45.045	G: ASP314, SER316, PHE317, TYR320; H: ARG107, ASP314; I: SER316		G: TYR320	G: ASN315, SER316, ASN321; H: HIS313, ASN315; I: THR312, SER316, THR322	G: PHE317, TYR320; I: LEU112, PHE317	G: ASP314; H: ASP314	H: ARG107; I: LYS113	
5	FLPLIGRVLSGIL	Temporin A	-2.787	2.406	G: ASP314, ASN315			G: ASN315, SER316, ASN321, HIS323; H: ASN321; I: ASN315, SER316	G: PHE317; H: PHE317, PRO318; I: LEU112, PHE317	G: ASP314		
6	FWQKMSFA	Plantaricin JLA-9	-9.002	-109.183	G: HIE313, ASP314; H: ASP314, ASN315, SER316; I: SER316	I: TYR320	H: ASP314	G: HIE313, ASN315, ASN321; H: ASN111, HIS313, ASN315, SER316, HIE323; I: SER316	G: PHE317; H: PHE317; I: PHE317, TYR320	G: ASP314; H: ASP314		
7	WWWLRKIW	TetraF2W-RK	-8.700	-122.832	G: ASP314, ASN315, ASN321; I: SER316	G: PHE317		G: ASN315, SER316, ASN321, HIS323; H: SER316; I: ASN111, SER316	G: LEU112, PHE317, PRO318, TYR320; H: PHE317; I: LEU112, PHE317, PRO318, TYR320	G: ASP314		G: GLY319
8	WNWSKSF	Darobactin	-9.605	-144.059	G: ASP314, ASN315; H: ASN315, SER316; I: ASN312		H: ASP314, PHE317	G: ASN315, ASN321; H: THR312, ASN315, SER316, HIE323; I: THR312, ASN315, SER316, ASN321, THR322	G: PHE317; H: PHE317; I: LEU112, PHE317	G: ASP314; H: ASP314		
9		MRL-494	-7.678	-66.449	G: ASP314; H: ASP314, ASN315;			G: ASN315; H: ASN315, HIE323; I: ASN111, THR322	G: LEU112; H: PHE317; I: PHE317, TYR320	G: ASP314; H: ASP314		

Sample Name :Plantaricin JLA-9
Sample ID :U9684GB030-1
Time Processed :11:14:02 AM
Month-Day-Year Processed :02/12/2021

Pump A : 0.065% trifluoroacetic in 100% water (v/v)
Pump B : 0.05% trifluoroacetic in 100% acetonitrile (v/v)
Total Flow:1 ml/min
Wavelength:220 nm
<<LC Time Program>>

Time	Module	Command	Value
0.01	Pumps	B.Conc	5
25.00	Pumps	B.Conc	65
25.01	Pumps	B.Conc	95
27.00	Pumps	B.Conc	95
27.01	Pumps	B.Conc	5
35.00	Pumps	B.Conc	5
35.01	Controller	Stop	

<<Column Performance>>
<Detector A>
Column :Inertsil ODS-3 4.6 x 250 mm
Equipment: ZJ17010508

<Chromatogram>

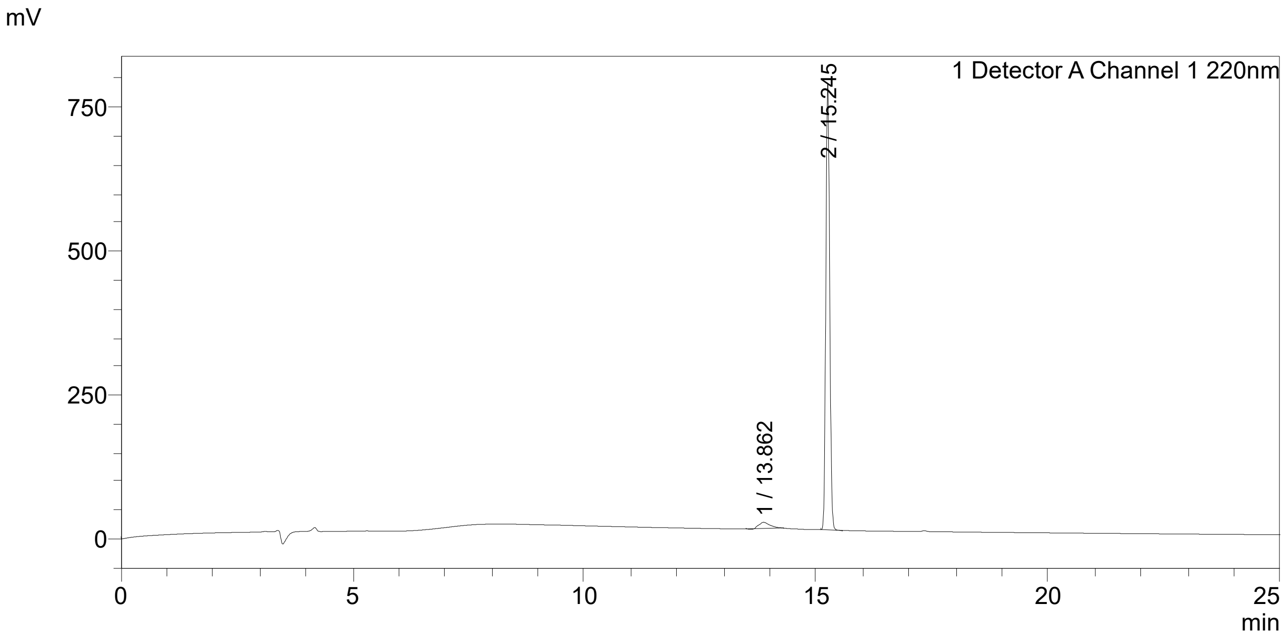

<Peak Table>

Detector A Channel 1 220nm

Peak#	Ret. Time	Area	Height	Area%
1	13.862	179955	10803	3.871
2	15.245	4469398	777168	96.129
Total		4649353	787971	100.000

Sample Name :Darobactin
Sample ID :U9684GB030-2
Time Processed :11:05:58
Month-Day-Year Processed :02/11/2021

Pump A : 0.065% trifluoroacetic in 100% water (v/v)
Pump B : 0.05% trifluoroacetic in 100% acetonitrile (v/v)
Total Flow:1 ml/min
Wavelength:220 nm
<<LC Time Program>>

Time	Module	Command	Value
0.01	Pumps	B.Conc	5
25.00	Pumps	B.Conc	65
25.01	Pumps	B.Conc	95
27.00	Pumps	B.Conc	95
27.01	Pumps	B.Conc	5
35.00	Pumps	B.Conc	5
35.01	Controller	Stop	

<<Column Performance>>
<Detector A>
Column :Inertsil ODS-SP 4.6 x 250 mm
Equipment: ZJ19010324

<Chromatogram>

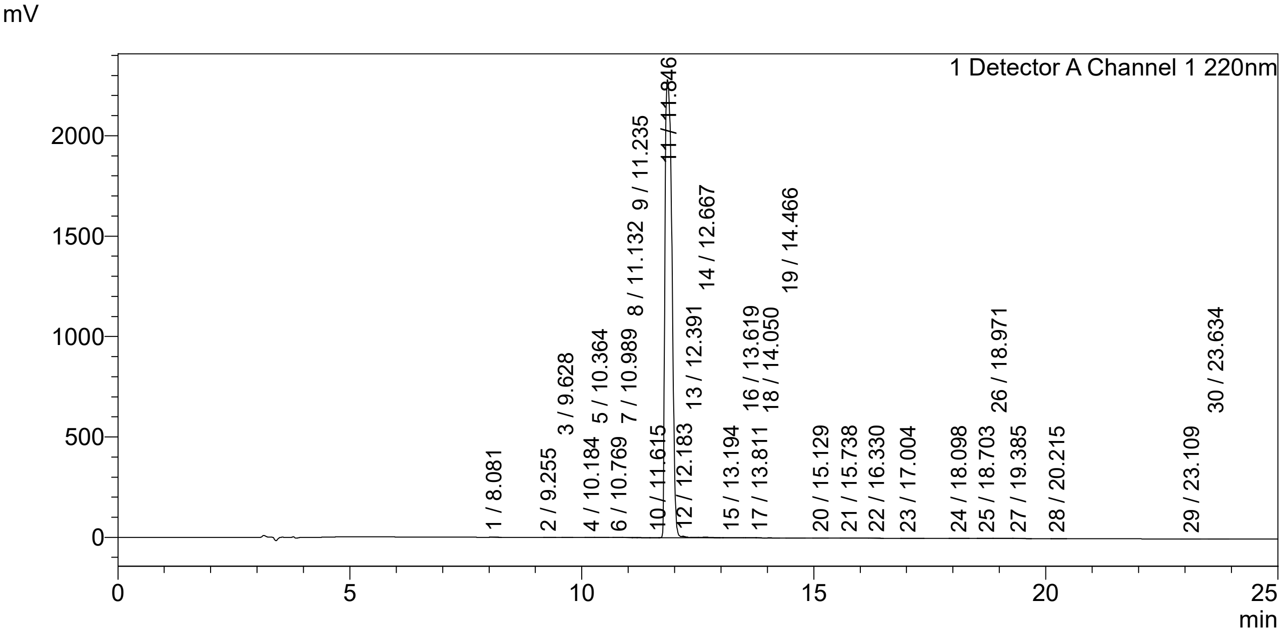

<Peak Table>

Detector A Channel 1 220nm

Peak#	Ret. Time	Area	Height	Area%
1	8.081	5715	1017	0.024
2	9.255	1141	134	0.005
3	9.628	1404	193	0.006
4	10.184	1945	364	0.008
5	10.364	2716	300	0.011
6	10.769	3391	633	0.014
7	10.989	5565	788	0.023
8	11.132	1860	331	0.008
9	11.235	4190	384	0.018
10	11.615	3393	419	0.014
11	11.846	23613171	2281339	99.455
12	12.183	7937	2054	0.033
13	12.391	1325	237	0.006
14	12.667	25287	2535	0.107

Peak#	Ret. Time	Area	Height	Area%
15	13.194	6559	467	0.028
16	13.619	1411	191	0.006
17	13.811	1364	305	0.006
18	14.050	2670	219	0.011
19	14.466	3890	232	0.016
20	15.129	3016	297	0.013
21	15.738	4859	194	0.020
22	16.330	2400	262	0.010
23	17.004	5224	264	0.022
24	18.098	3199	349	0.013
25	18.703	9326	636	0.039
26	18.971	8209	579	0.035
27	19.385	5892	537	0.025
28	20.215	1290	156	0.005
29	23.109	2631	165	0.011
30	23.634	1516	114	0.006
Total		23742499	2295693	100.000

Sample Name :Urechistachykinin II
Sample ID :U9684GB030-3
Time Processed :10:14:50
Month-Day-Year Processed :02/13/2021

Pump A : 0.065% trifluoroacetic in 100% water (v/v)
Pump B : 0.05% trifluoroacetic in 100% acetonitrile (v/v)
Total Flow:1 ml/min
Wavelength:220 nm
<<LC Time Program>>

Time	Module	Command	Value
0.01	Pumps	B.Conc	5
25.00	Pumps	B.Conc	65
25.01	Pumps	B.Conc	95
31.00	Pumps	B.Conc	95
31.01	Pumps	B.Conc	5
40.00	Pumps	B.Conc	5
40.01	Controller	Stop	

<<Column Performance>>
<Detector A>
Column :Inertsil ODS-3 4.6 x 250 mm
Equipment: ZJ19010140

<Chromatogram>

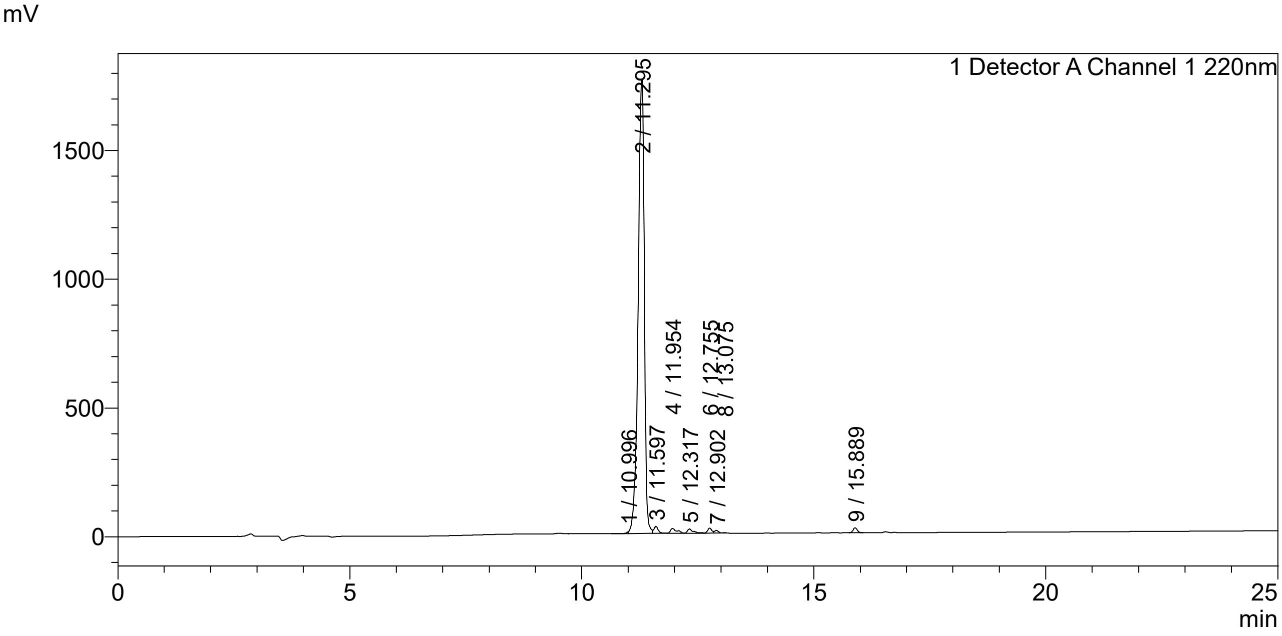

<Peak Table>

Detector A Channel 1 220nm

Peak#	Ret. Time	Area	Height	Area%
1	10.996	16731	6379	0.098
2	11.295	16229330	1763626	95.008
3	11.597	181365	27337	1.062
4	11.954	187117	18934	1.095
5	12.317	149772	16880	0.877
6	12.755	125736	19862	0.736
7	12.902	61962	10218	0.363
8	13.075	10164	2092	0.059
9	15.889	119867	19090	0.702
Total		17082045	1884418	100.000
